# 血液肿瘤双特异性抗体药物的临床研究进展

**DOI:** 10.3760/cma.j.issn.0253-2727.2023.05.015

**Published:** 2023-05

**Authors:** 奕茗 蔡, 德沛 吴, 杨 徐

**Affiliations:** 苏州大学附属第一医院，国家血液系统疾病临床医学研究中心，江苏省血液研究所，卫生部血栓与止血重点实验室，苏州大学造血干细胞移植研究所，苏州 215006 The First Affiliated Hospital of Soochow University; National Clinical Research Center for Hematologic Diseases; Jiangsu Institute of Hematology; Key Laboratory of Thrombosis and Hemostasis Under Ministry of Health; Institute of Blood and Marrow Transplantation, Soochow University, Suzhou 215006, China

双特异性抗体（BsAb，简称“双抗”）是一类可特异性结合两种抗原或同一抗原不同表位的人工抗体，经基因工程、细胞融合或化学偶联制备。双抗可介导免疫细胞杀伤肿瘤，或结合双重肿瘤抗原调节多条通路，或识别免疫细胞不同免疫检查点进行免疫调控，具有广泛临床应用前景。随着血液肿瘤药物贝林妥欧单抗（Blinatumomab）上市，双抗研发取得成果同时也面临挑战。本文对双抗药物治疗血液肿瘤的临床研究进展进行综述。

一、概述

20世纪60年代，美国免疫学家Nisonoff将两种血清免疫球蛋白的抗原结合片段（Fab）经再氧化连接，所得产物成功募集两种相应抗原，双抗的概念被首次提出[1]。1983年，继应用杂交瘤细胞制备单克隆抗体后，作为潜在治疗方法的双抗也获得关注，Milstein与Cuello[2]创造性融合两种杂交瘤细胞系，成功获得分泌双抗的四源杂交瘤细胞。1985年，双抗临床意义初现，Staerz等[3]首次制备出可募集T细胞攻击肿瘤细胞的双抗，为介导免疫细胞杀伤靶细胞的抗体研发奠定基础。20世纪90年代，双抗治疗恶性肿瘤的临床试验兴起，但广泛应用的四源杂交瘤法存在“链交联问题”，即抗体重链、轻链随机配对导致错配抗体大量产出、目标双抗产率低下，亟需更为成熟的批量化生产方式。1996年Ridgway等[4]在两异源抗体重链恒定区引入突变以形成“杵臼结构（Knob-into-Holes, KiH）”，使异源重链正确装配，有效提高双抗产出。随着细胞工程、基因工程技术不断进步，除KiH外也出现更多改造抗体的方法，共同解决或直接避免链交联问题，并改善双抗强免疫原性、低稳定性等不足，许多方法已成功转化为商业化生产平台。

不同平台生产的双抗总体分为两类：IgG样抗体与非IgG样抗体。天然IgG抗体为两条相同重链与相同轻链连接形成的Y形单体，具有两个Fab片段和一个可结晶片段（Fc）。IgG样双抗由两种不同特异性的单价抗体拟Y形态组合形成，含有Fc片段，分子量大，半衰期长，也保留了Fc片段相应功能，如抗体依赖的细胞介导的细胞毒性、补体依赖的细胞毒性、抗体依赖的细胞介导的吞噬作用。KiH、CrossMab、Duobody等均是该类双抗的技术平台。非IgG样双抗由不同特异性的抗体片段连接形成，不含Fc片段，无其介导的相关功能，主要通过结合有关抗原发挥作用。该类双抗分子量小，组织渗透性高但半衰期短，临床应用时常需持续给药。双特异性T细胞衔接器（Bispecific T-cell engagers，BiTE）是代表性非IgG样双抗，由识别T细胞CD3分子与靶细胞抗原的两种单链可变区（Single-chain variable fragment，scFv）片段连接形成，通过募集肿瘤组织附近的多克隆T细胞群、衔接肿瘤细胞而高效发挥抗肿瘤效应。TandAb、DART等为同属该类双抗的技术平台。迄今已有超100种技术平台制备性能各异的双抗，为临床应用提供多样化选择。

二、临床应用

（一）淋系肿瘤

1. CD19/CD3：

CD3仅存于T淋巴细胞表面，与T细胞抗原识别受体（TCR）跨膜带氨基酸残基形成盐桥，转导TCR识别抗原的活化信号，激活T细胞；CD19是B淋巴细胞表面蛋白特异性标志，在B细胞共受体识别抗原中起到信号转导作用并调控B细胞增殖分化，是B细胞肿瘤治疗的理想靶点。靶向CD19/CD3双抗现用于前体B细胞急性淋巴细胞白血病（BCP-ALL）与B细胞非霍奇金淋巴瘤（B-NHL）治疗。

（1）贝林妥欧单抗：贝林妥欧单抗基于BiTE平台开发的首个全球范围内上市的血液肿瘤双抗，可分别结合T细胞表面的CD3分子与B细胞CD19表面抗原并在其间形成突触，通过细胞毒作用持续裂解靶细胞[5]。一项早期Ⅱ期临床试验验证了贝林妥欧单抗在Ph阴性复发难治（R/R）BCP-ALL患者中的疗效与安全性，189例成人患者经两周期贝林妥欧单抗单药治疗后完全缓解（CR）率达33％，并可持续6.9个月，2％的患者发生3级细胞因子释放综合征（CRS）[6]。2014年FDA基于该试验加速批准贝林妥欧单抗用于治疗成人Ph阴性R/R BCP-ALL。研究者为进一步探究贝林妥欧单抗对过度治疗的Ph阴性BCP-ALL疗效启动了多中心、随机对照的Ⅲ期TOWER研究，结果表明贝林妥欧单抗单药治疗组CR率（33％对16％，*P*<0.001）与微小残留病（MRD）阴性率（76％对48％）显著高于标准化疗组，中位总生存（OS）期延长（7.7个月对4.0个月，*HR*＝0.71，*P*＝0.01）[7]。此外，Topp等[8]长期追踪贝林妥欧单抗治疗后缓解的R/R BCP-ALL患者发现，两周期疗程内即获CR或完全缓解伴部分血液学恢复（CRh）患者的3年无复发生存（RFS）率高达59.3％；56例桥接造血干细胞移植术的CR/CRh者中位OS期为18.1个月，3年OS率为37.2％。这些数据表明贝林妥欧单抗可为更多患者争取桥接移植机会并使其长期获益。

不限于Ph阴性患者，Ⅱ期ALCANTARA研究将贝林妥欧单抗用于45例Ph阳性R/R BCP-ALL患者，其均曾使用至少1种二代或更高级别酪氨酸激酶抑制剂，16例患者获得CR/CRh，其中88％ MRD阴性、44％桥接移植，CR/CRh者中位RFS与OS期分别为6.7与7.1个月[9]。2017年FDA基于ALCANTARA与TOWER研究给予贝林妥欧单抗上市的完全批准，并将适应证扩大至Ph阳性的R/R BCP-ALL治疗。

除形态学缓解外，贝林妥欧单抗能够有效清除MRD，使患者获得更深度的分子学缓解。2018年贝林妥欧单抗被FDA批准用于治疗首次或二次CR伴MRD阳性的成人及儿童BCP-ALL。该批准主要基于Ⅱ期BLAST研究，116例CR伴MRD阳性的成人BCP-ALL患者经一周期治疗后MRD阴性率达78％，其中110例Ph阴性患者18个月RFS率为54％，OS期为36.5个月，74例（67％）桥接造血干细胞移植；治疗后MRD阴性者生存获益显著（RFS：23.6个月对5.7个月，*P*＝0.002；OS：38.9个月对12.5个月，*P*＝0.002）[10]。提示应用贝林妥欧单抗作为MRD清除治疗能有效改善患者预后。

此外达沙替尼诱导缓解联合贝林妥欧单抗巩固治疗新发Ph阳性BCP-ALL[11]及Blinatumomab巩固治疗再诱导后的儿童、青少年及年轻成人Ph阴性BCP-ALL患者均取得成效[12]。这些研究表明早期应用贝林妥欧单抗或可减少化疗需求，并使患者生存获益更多。现还有临床试验研究该药联合新型CD22靶向药物Inotuzumab Ozogamicin治疗初诊B-ALL患者（NCT03739814），贝林妥欧单抗更多应用方案正探索中。

（2）AFM11：AFM11为包含四个scFv结构域的四价TandAb类双抗，因此分子量较贝林妥欧单抗大、半衰期长、亲和力高。体外实验中AFM11对CD19阳性肿瘤细胞具有良好的抗肿瘤活性[13]，目前已进入Ⅰ期临床试验。

2. CD20/CD3：

CD20表达于浆细胞外的各阶段B细胞，通过调节钙离子跨膜流动影响B细胞增殖分化，是B细胞系肿瘤靶向药物识别的靶分子，利妥昔单抗联合高剂量化疗与自体造血干细胞移植（ASCT）是治疗R/R侵袭性B-NHL的主要方法，但许多患者无法耐受高强度化疗；惰性滤泡淋巴瘤（FL）虽进展缓慢，但患者面临多次复发乃至转化为侵袭性NHL的问题。CD20/CD3双抗成为R/R B-NHL的新疗法。

（1）Mosunetuzumab：Mosunetuzumab为基于KiH平台开发的IgG1样双抗，已于2022年6月获得欧盟附条件上市批准，用于既往至少接受过2线治疗的成人R/R FL。该批准基于Ⅰ/Ⅱ期GO29781研究，试验中Mosunetuzumab单药治疗既往接受过2线及以上治疗方案的R/R FL患者ORR达78.9％，CR率达57.8％，中位无进展生存（PFS）期为17.9个月，90例入组患者仅2例出现3级及以上CRS[14]。该研究表明Mosunetuzumab单药治疗R/R FL具有良好的疗效与可靠的安全性。除单一用药外，Ⅰb研究观察到来那度胺联合Mosunetuzumab治疗的13例R/R FL患者中10例获得完全代谢缓解（CMR），高级别不良事件发生率为30％（8/27），该Ⅰb研究初步证实来那度胺联合方案有效、安全可控[15]，其结果支持启动随机对照、多中心Ⅲ期CELESTIMO研究，以进一步评估该方案并同利妥昔单抗联合来那度胺治疗R/R FL作出比较。此外，对于R/R弥漫大B细胞淋巴瘤（DLBCL）等侵袭性NHL，Mosunetuzumab单药治疗时患者ORR与CR率分别为34.9％与19.4％[16]。

（2）Glofitamab：Glofitamab为CrossMab平台的IgG1样、“2∶1”特殊结构双抗，存在2个CD20结合域与1个CD3结合域，比其他“1∶1”结构抗体亲和力更强，具备联用抗CD20单抗、提高疗效的潜能。Glofitamab对侵袭性B-NHL疗效较Mosunetuzumab好。2022年美国临床肿瘤学会（ASCO）会议更新了NP30179研究Ⅱ期试验结果，107例R/R DLBCL患者中59％原发难治、32％CAR-T治疗失败，在Glofitamab首剂治疗前予奥妥珠单抗预处理以降低CRS风险，ORR与CR率分别为50.0％与35.2％，84％的CR持续至少9个月，且既往接受嵌合抗原受体T细胞（CAR-T）疗法与未接受CAR-T疗法的患者CR率相当（32％对37％）；3级（3％）、4级（2％）CRS罕见，3例神经系统事件均为低级别[17]。该Ⅱ期临床试验结果进一步证实安全性高的Glofitamab能诱导R/R DLBCL患者产生持久缓解，并对既往应用CAR-T疗法的患者有效，临床疗效显著。

（3）Epcoritamab：Epcoritamab是DuoBody平台的IgG1样双抗。Ⅰ/Ⅱ期剂量递增试验的EPCORE NHL-1研究[18]显示22例12～60 mg给药的DLBCL患者ORR与CR率分别为68％、45％，10例0.76～48 mg给药的FL患者ORR与CR率分别为90％、50％，未发生3级及以上CRS。另一项评估Epcoritamab联合用药治疗B-NHL的Ⅰ/Ⅱ期EPCORE NHL-2研究在多个试验组中观察到了积极的结果。试验组1旨在评估Epcoritamab联合R-CHOP作为一线治疗方案用于初诊DLBCL的疗效及安全性，其中24％为双打击或三打击DLBCL，所有患者ORR为94％（24/25），完成6周期疗程的10例患者ORR与CMR率分别达到100％与90％，CRS多为低级别[19]，初步表明Epcoritamab联合R-CHOP一线治疗初诊DLBCL有效、安全可行。试验组2中27例联合来那度胺治疗的R/R FL患者ORR为100％，CMR率为93％，中位随访5.0个月，患者均持续缓解[20]。试验组4中23例R/R DLBCL患者联合利妥昔单抗、地塞米松、阿糖胞苷、奥沙利铂或卡铂（R-DHAX/C）方案治疗后ORR达83％，CMR率为61％，11例桥接ASCT[21]。EPCORE NHL-1与EPCORE NHL-2研究共同表明Epcoritamab单药治疗R/R NHL时缓解率高、安全性好，联合用药也具有前景。现Epcoritamab单药治疗R/R DLBCL与化疗比较的随机对照、多中心Ⅲ期临床试验（NCT04628494）已经启动。

（4）奥妥珠单抗（Odronextamab）：奥妥珠单抗为IgG4样、靶向CD20/CD3双抗药物，对CAR-T难治的B-NHL展现出一定疗效与可接受的安全性。Ⅰ期剂量递增试验显示142例过度治疗的R/R B-NHL患者客观缓解率为51％，且30例曾接受CAR-T疗法的DLBCL患者中8例（27％）获得CR，CRS与神经系统事件多为低级别，但4例发生治疗相关死亡[22]。Ⅱ期临床试验正进一步评估奥妥珠单抗的疗效与安全性。

3. CD30/CD16A：

CD16A表达于NK细胞、巨噬细胞与单核细胞表面，是激活NK细胞的受体；CD30分子高表达于霍奇金淋巴瘤（HL）R-S细胞表面而在正常细胞中表达有限。AFM13为NK细胞靶向双抗中进展最快的双抗，为靶向CD30/CD16A的TandAb，可结合CD16A激活和募集NK细胞杀伤CD30阳性肿瘤。

Ⅰ期剂量递增试验显示AFM13治疗R/R HL的疾病控制率为61.5％，并对本妥昔单抗（Brentuximab）耐药患者有一定疗效[23]。Kerbauy等[24]体外实验中发现AFM13联合细胞因子激活的外周血或脐血来源的NK细胞可更高效地杀伤CD30阳性肿瘤细胞，他们因此开展AFM13联合脐血来源的NK细胞治疗CD30阳性R/R HL或NHL的Ⅱ期临床试验（NCT04074746），有望进一步改善患者预后。

（二）浆细胞病

1. BCMA/CD3：B细胞成熟抗原（BCMA）为肿瘤坏死因子受体超家族成员，因其选择性表达于B细胞表面并在肿瘤细胞中过表达而成为治疗MM的靶分子。Teclistamab为靶向BCMA/CD3的DuoBody双抗，已经被FDA授予治疗复发难治性多发性骨髓瘤（RRMM）的突破性药物资格。Ⅰ/Ⅱ期MajesTEC-1研究纳入165例既往接受至少3线治疗的RRMM患者，中位随访14.1个月，ORR与CR率分别为63.0％与39.4％，共44例获MRD阴性，总体中位PFS期为11.3个月；常见不良事件有CRS（72.1％，3级0.6％，无更高级别发生）、中性粒细胞减少等，出现5例（3％）免疫细胞效应相关神经毒性综合征[25]。Teclistamab安全性高并给多线治疗失败的MM患者带来深度、持久的缓解，已进入Ⅲ期临床试验。其他BCMA/CD3双抗AMG420、REGN5458等也在临床研究中。

2. GPRC5D/CD3：GPRC5D属于G蛋白偶联孤儿受体，高度选择性表达于MM细胞且在正常组织中很少表达。Talquetamab是采用DuoBody平台开发的靶向GPRC5D/CD3双抗。Ⅰ期剂量递增试验表明30例以每周405 µg/kg给药的RRMM患者ORR为70％，以每两周800 µg/kg给药的18例患者ORR为71％，中位持续缓解时间未达；常见不良事件为CRS（1例3级，无更高级别发生）、中性粒细胞减少、味觉障碍等[26]。Talquetamab现进入Ⅱ期临床试验。

3. FcRH5/CD3：FcRH5几乎在所有骨髓瘤细胞上表达且表达率显著高于正常B细胞。Cevostamab是靶向FcRH5/CD3双抗，对于过度治疗的RRMM患者展初步现出良好的活性。在一项Ⅰ期剂量递增试验中，目标剂量为160 mg时RRMM患者ORR为54.5％（24/44），目标剂量>90 mg时既往使用CAR-T疗法、其他双抗、抗体偶联药物、抗BCMA治疗的患者ORR分别达44.4％（4/9）、33.3％（3/9）、50.0％（7/14）与36.4％（8/22）[27]。

（三）髓系肿瘤

1. CD123/CD3：CD123在急性髓系白血病（AML）、母细胞性浆细胞样树突细胞肿瘤等血液肿瘤中广泛表达，与IL-3结合诱导细胞增殖与活化。Flotetuzumab是靶向CD123/CD3的DART双抗，一项Ⅰ/Ⅱ期临床评估了Flotetuzumab单药治疗初始诱导失败或早期复发AML患者的安全性及疗效，Ⅱ期推荐剂量治疗时CR/CRh率为26.7％（8/30），CR/CRh者中位OS期为10.2个月；3级注射相关反应或CRS发生率为8.0％[28]。

2. CD33/CD3：CD33以跨膜蛋白形式存在于正常髓系前体细胞、成熟粒细胞与单核细胞上表面，并在大量AML细胞上表达。AMG330是靶向CD33/CD3的BiTE双抗，在Ⅰ期临床试验中35例过度治疗的AML患者2例获得CR，1例获得血液学不完全恢复的CR（CRi）[29]。

三、不足

（一）肿瘤细胞免疫逃逸

1. 免疫检查点激活：Krupka等[30]发现在原发AML细胞样本中AMG330介导的T细胞活化明显上调程序性死亡配体1（PD-L1）与程序性死亡受体1（PD-1）表达，而抑制PD-1/PD-L1后AMG330裂解AML细胞增强。该研究揭示应用AMG330时引起细胞因子大量释放，可能诱发AML肿瘤细胞上调PD-L1表达以进行免疫逃逸。联合免疫检查点抑制剂、开发靶向免疫检查点的双抗是提高疗效的潜在策略。

2. 抗原丢失变异：仅针对1个肿瘤抗原可能发生靶抗原丢失乃至药物耐药。潜在解决方法为开发靶向不同肿瘤抗原的三特异性或多特异性抗体。

（二）肿瘤微环境（TME）抑制

TME可通过物理屏障减少肿瘤浸润淋巴细胞（TIL），或利用髓系来源抑制细胞、Treg细胞、抑制性细胞因子等降低免疫细胞活性。现多数双抗依赖免疫细胞介导的抗肿瘤效应发挥作用，肿瘤组织附近的TIL水平或影响双抗疗效[31]。

（三）临床应用问题

1. 给药方式不便：短效非IgG样抗体应用时常需持续给药，以贝林妥欧单抗为例，每个疗程建议包括4周持续静脉输射，造成注射部位不良反应。现有创新性研究利用溶瘤病毒、转染T细胞或转染间充质细胞作为载体于肿瘤组织原位释放双抗以克服持续给药不便、改善TME免疫抑制[32]。

2. 不良反应：CRS是常见不良事件，因IL-6在内的细胞因子大量释放造成。以贝林妥欧单抗为例预防性应用地塞米松、递增剂量给药可降低CRS发生率，贝林妥欧单抗应用后可能发生震颤等神经系统不良反应，但相关机制尚不明确，多数停药、应用地塞米松后症状可逆。

四、展望

免疫治疗发展迅速，双抗与CAR-T疗法各有利弊。即用型双抗无需历经收集、改造患者免疫细胞的繁琐步骤，是疾病进展快速时便捷的选择；而CAR-T细胞具有自我扩增能力，生命周期更加持久。现双抗仍面临选择新靶点、高效激活效应细胞、克服免疫抑制微环境、减少不良反应等挑战，期待双抗未来或带来更多突破，改变血液肿瘤治疗模式。
